# Supplementary material for: The Interplay Between Splicing of Two Exon Combinations Differentially Affects Membrane Targeting and Function of Human CaV2.2
Source: Function (Oxf). 2023 Oct 19;5(1):zqad060. doi: 10.1093/function/zqad060 (PMC10666670; doi:10.1093/function/zqad060)
Supplement: zqad060_Supplemental_Files [file zqad060_supplemental_files.zip › Dahimene et al Supplementary Figures_rev_171023.docx]

**Supplementary Figures**

**The interplay between splicing of two exon combinations differentially affects membrane targeting and function of human Ca_V_2.2**

Shehrazade Dahimene*, Karen M Page*, Manuela Nieto-Rostro*, Wendy S Pratt and Annette C Dolphin.

**Supplementary Figure 1**

**
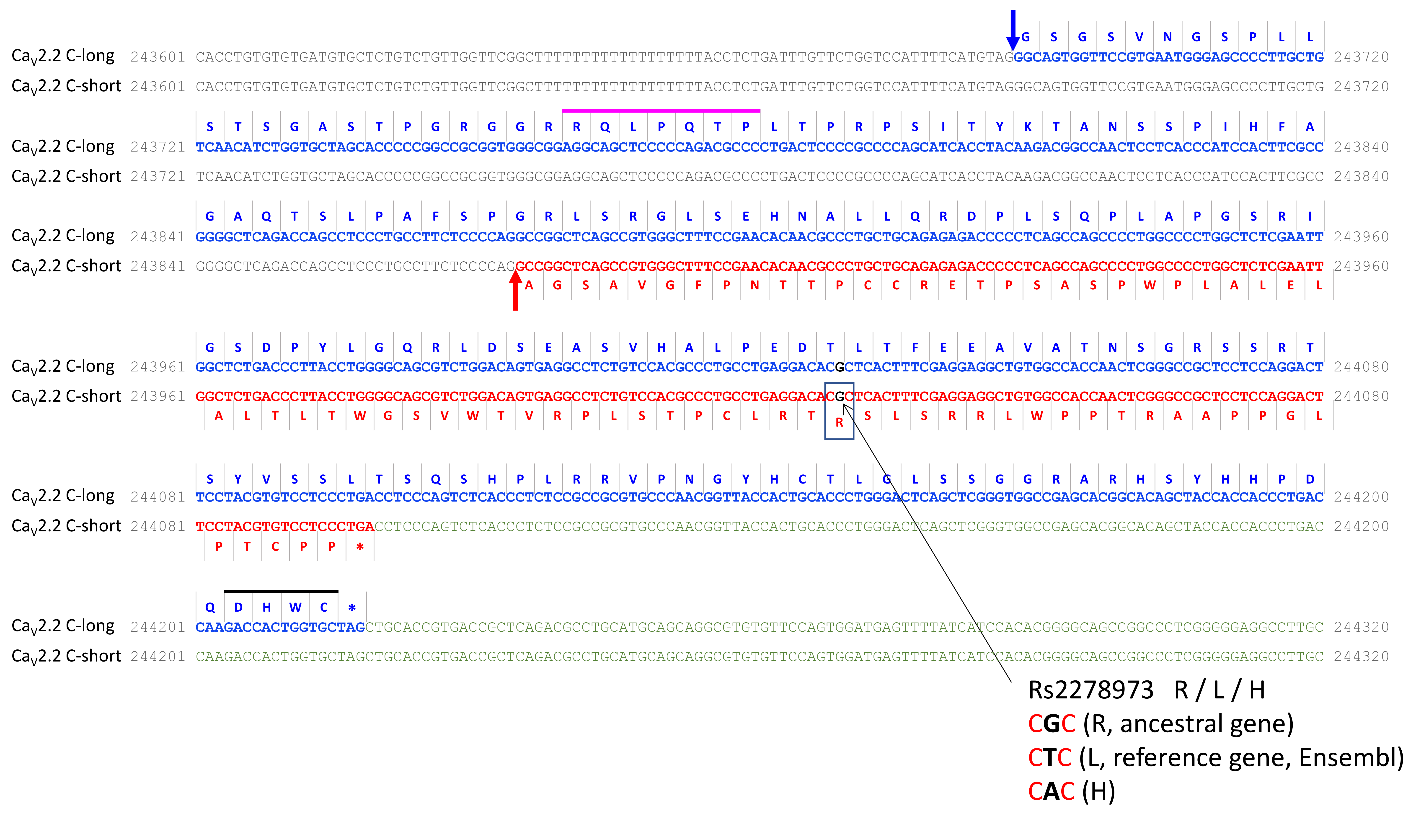
**

**Non-synonymous SNPs in short exon47 of human *CACNA1B***

DNA sequences for the Ca_V_2.2 variants having the long and short C termini. Alternative splice sites are indicated by arrows, blue for the long variant, red for the short. Amino acid sequences are shown in blue for the long variant (above sequence) and red for the short variant (below sequence). Intron sequence is in grey and 3’ untranslated region is in green. The in-frame stop codons for the two variants are indicated by *. The Rs2278973 snp is synonymous in the long variant (ACG/ACT/ACA = T) but results in an amino acid change (CGC=R, CTC=L, CAC=H) in the short variant. According to the gnomAD genomes v3.1.2 database (*Ensembl*), the variants have the following world-wide frequency count: Arg (CGC): 91.7%, Leu (CTC): 8.27%, His (CAC): 0.003%. The proline-rich SH3-binding domain (which bind SH3 domains) was identified previously to bind RIM binding protein ^11^ and is shown as a pink line above the sequence; the PDZ-interacting domain, DXWC ^6,10^ at the end of the C-long sequence, is shown as a black line. **Supplementary Figure 2**

**
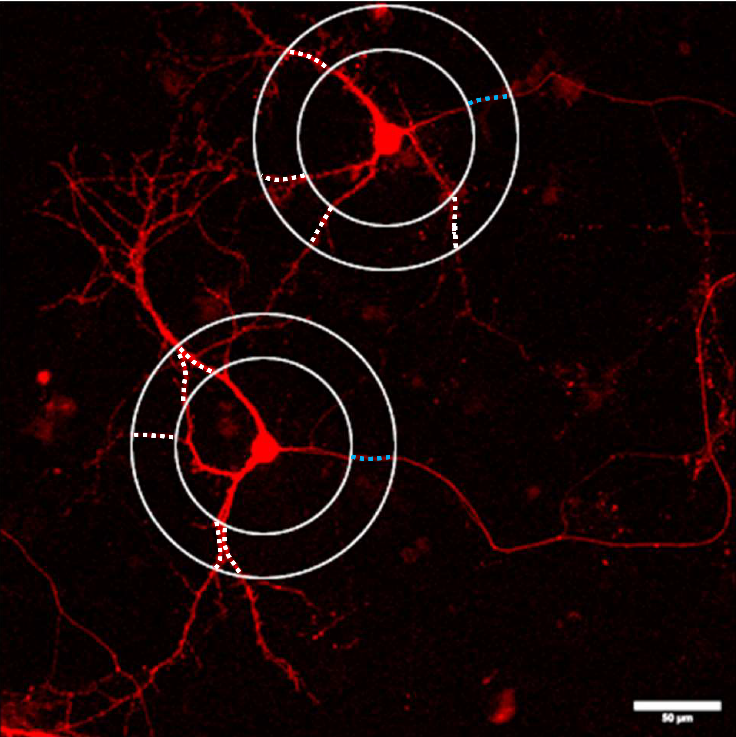
**

**Example of ROI selection for analysis of hipocampal neurites**

Confocal image of hippocampal neurons using 20× objective with a 5 μm optical section. The fluorescence intensity along neuronal projections was assessed as follows: two concentric circles of 100 μm and 150 μm diameter were drawn around each neuronal cell body. A freehand line tool of 3 μm width tracing the neuronal dendrites (3 to 5 averaged per neuron, white dotted line) and axons (one per neuron, blue dotted line, identified by smooth morphology, lack of branching and narrow diameter) was drawn between the circles in the mCherry images (shown) and used as template for GFP and HA images. Scale bar 50 µm.

**Supplementary Figure 3**

**
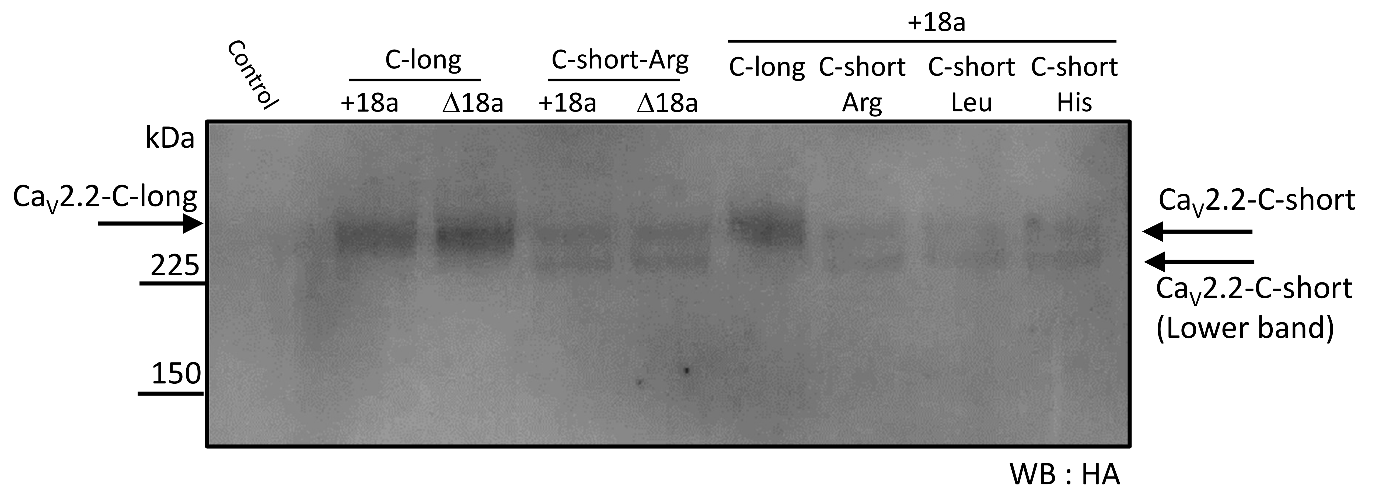
**

**Lack of effect of Ca_V_2.2 C-short SNP variants on total Ca_V_2.2 protein expression**

Full immunoblot of whole-cell lysates (WCL) from tsA-201 cells transfected, as stated, with either GFP_Ca_V_2.2-HA C-long, C-short (Arg) (with or without exon18a), C-short-Leu or C-short-His (both + exon18a), together with α_2_δ-1 and β1b. Arrows indicate GFP_Ca_V_2.2-HA C-long (left) and GFP_Ca_V_2.2-HA C-short (right, 2 bands). Immunoblot was performed using anti-HA antibody. Partial blot shown in Fig. 5D.

**Supplementary Figure 4**

**
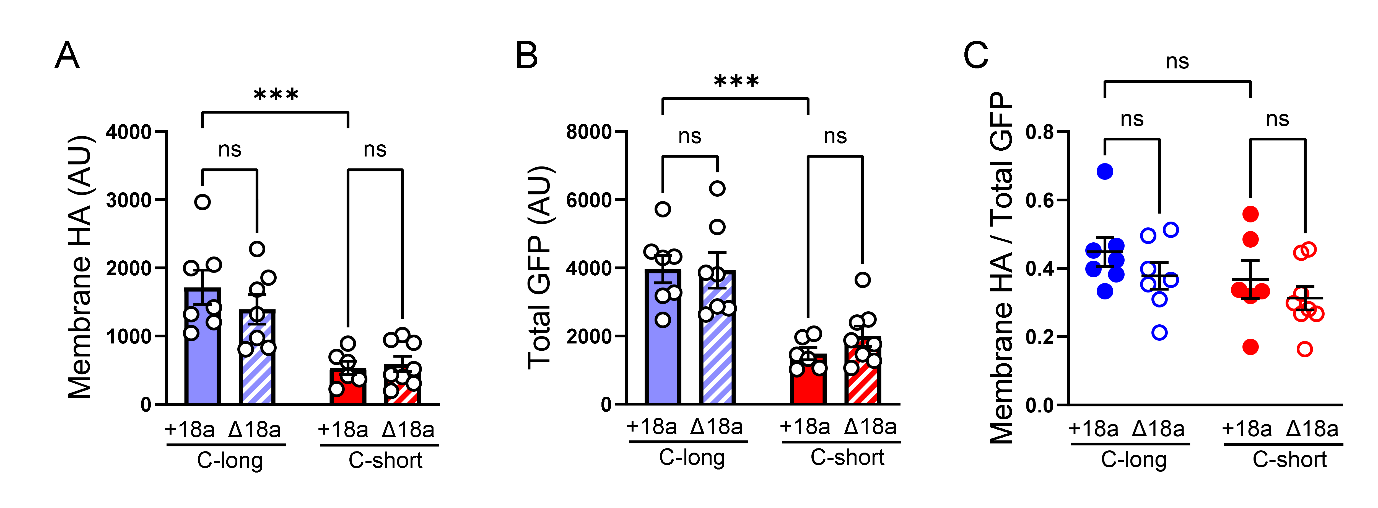
**

**Effects of long or short C-terminal exon47 with or without exon18a on Ca_V_2.2 expression in axons of hippocampal neurons**

Quantification of Ca_V_2.2 at the membrane (HA, A) or total (GFP, B) and the ratio of HA/GFP (C) in hippocampal neuron axons, identified by length, lack of branching and small diameter (see Supplementary Fig. 2 for examples). Data are taken from the same experiments as in Figure 4, and are quantified as described for Figure 4D. Bars show mean ± SEM, and each dot represents the mean value for each coverslip. Statistical significance is indicated with *** P<0.001, and ns: non-significant (two-way ANOVA followed by Bonferroni’s multiple comparisons test; see Supplementary Data 1 for source of variations and interactions between groups).

**Supplementary Figure 5**

**
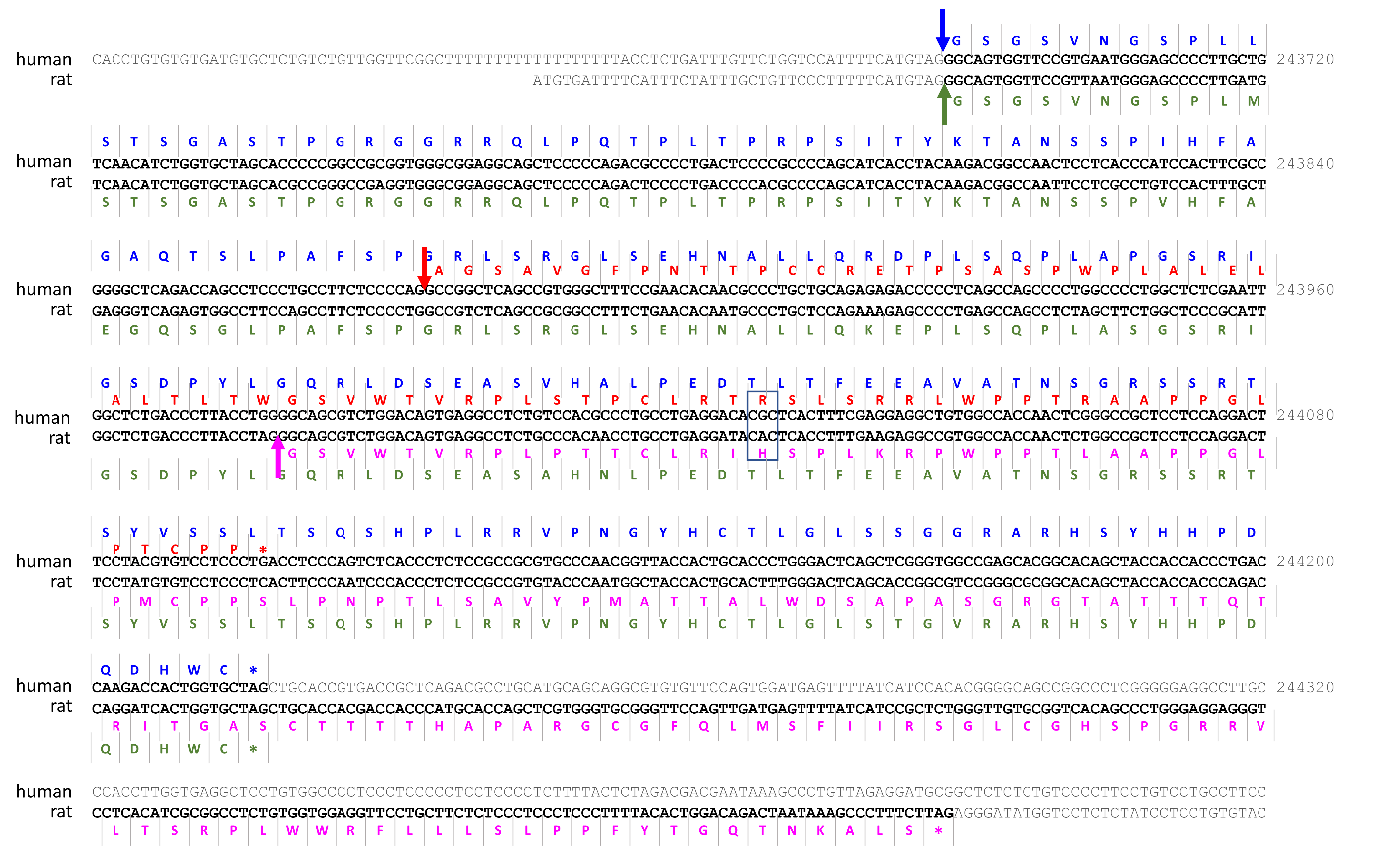
**

**Comparison of organization of rat and human *CACNA1B* transcripts and amino acid sequences**

DNA sequences are shown for the Ca_V_2.2 variants having the long and short C termini in human and rat. Alternative splice sites are indicated by arrows, blue for the human long variant, red for the human short, green for the rat long and pink for the rat short. Amino acid sequences are shown in blue for the human long and red for the human short variants (above sequence) and green for the rat long and pink for the rat short variants (below sequence). Intron sequence and 3’ untranslated region is in grey. The in-frame stop codons are indicated by *.
